# Utility of Circulating Tumor DNA in Different Clinical Scenarios of Breast Cancer

**DOI:** 10.3390/cancers12123797

**Published:** 2020-12-16

**Authors:** Alexandra Mesquita, José Luís Costa, Fernando Schmitt

**Affiliations:** 1 Medical Oncology Department, Hospital Pedro Hispano, Unidade Local Saúde Matosinhos, 4464-513 Senhora da Hora, Portugal; 2 Institute of Molecular Pathology and Immunology, University of Porto, 4200-135 Porto, Portugal; jcosta@ipatimup.pt (J.L.C.); fschmitt@ipatimup.pt (F.S.); 3 Faculty of Medicine, University of Porto, 4200-319 Porto, Portugal

**Keywords:** ctDNA, breast cancer, clinical utility, molecular biology, next generation sequencing

## Abstract

**Simple Summary:**

This review is focused on the concept of a specific type of “liquid biopsy”, circulating cell-free tumor DNA (ctDNA). It explores the advantages and limitations of using this technique and the latest advances of using it in different clinical scenarios of breast cancer: early, metastatic, and locally advanced disease. It provides the latest advances in this area applied to clinical research and clinical practice, as well as the importance of the collaboration between clinicians and laboratory teams to fully grasp the potential of ctDNA in a precision medicine era.

**Abstract:**

Breast cancer is a complex disease whose molecular mechanisms are not completely understood. Developing target therapies is a promising approach. Therefore, understanding the biological behavior of the tumor is a challenge. Tissue biopsy in the metastatic setting remains the standard method for diagnosis. Nevertheless, it has been associated with some disadvantages: It is an invasive procedure, it may not represent tumor heterogeneity, and it does not allow for treatment efficacy to be assessed or early recurrences to be detected. Analysis of circulating tumor DNA (ctDNA) may help to overcome this as it is a non-invasive method of monitoring the disease. In early-stage disease, it can detect early recurrences and monitor tumors’ genomic profiles, identifying the emergence of new genetic alterations which can be related to tumor-acquired resistance. In the metastatic setting, the analysis of ctDNA may also allow for the anticipation of clinical and radiological progression of the disease, selection of targeted therapies, and for a photogram of tumor heterogeneity to be provided. It may also detect disease progression earlier in locally advanced tumors submitted to neoadjuvant treatment, and identify minimal residual disease. ctDNA analysis may guide clinical decision-making in different scenarios, in a precision medicine era, once it acts as a repository of genetic tumor material, allowing for a comprehensive mutation profiling analysis. In this review, we focused on recent advances towards the implementation of ctDNA in a clinical routine for breast cancer.

## 1. Introduction

Breast cancer is the most prevalent cancer in women worldwide [1]. Regarding cancer-related mortality, it is the second cause of death in developed regions [1]. The incidence has increased since the introduction of mammography screening, and continues to grow with the ageing of the population [2].

Tissue biopsy of the tumors remains the standard method for cancer diagnosis. Nevertheless, it has been associated with several disadvantages in metastatic and recurrent breast cancer cases: It may not represent the tumor heterogeneity and it does not allow for the evaluation of treatment efficacy or detection of early recurrences or residual disease [3,4]. In addition, it cannot be used repeatedly due to the invasiveness of the procedure for the patient, cost, and time consumption [5], and so it cannot be used for monitoring the disease along time.

Therefore, new methodologies of studying the dynamic range of molecular alterations of the tumor have been developed. Recent findings in DNA sequencing and digital molecular techniques have supported the clinical use of circulating tumor DNA (ctDNA) as a “liquid biopsy” [6]. Liquid biopsies have emerged in a precision medicine era and they, already, play an important role in the decision making process in some cancers such as lung and colorectal cancer [7,8]. In breast cancer patients, clinical, histological, and immune-histochemical features of the tumor have guided the most appropriate treatment approaches for years. Breast cancer tumors are classified as luminal A, luminal B, luminal B Her2-positive, and triple-negative breast cancers according to the presence of estrogen and progesterone receptors, and HER2 high positivity or values of proliferation marker ki67 [9]. This classification helps to guide medical oncologists to choose the most appropriate treatment.

The heterogeneity of the breast cancer disease and variations between clinical and molecular classification was addressed by Dawson, S.J. et al. A classification based on molecular information on the genomic and transcriptomic landscapes of breast cancer was able to define 10 molecular clusters associated with different clinical outcomes. The integrated analysis of the somatic copy number aberrations (CNAs) and their effect on gene expression allowed novel molecular subgroups to be identified and, therefore, a better clarification of breast cancer heterogeneity [10].

In recent years, molecular characterization and identification of mutations of the tumors have gained more relevance in breast cancer patients, mainly in the metastatic disease, due to the development of targeted therapies.

## 2. ctDNA—The Concept

Circulating cell-free DNA (cfDNA) was firstly reported in 1948 in the blood of healthy people [11], and later in cancer patients [12].

Cell-free DNA is fragmented DNA that is found in the non-cellular blood components of healthy individuals. Among tumor patients, ctDNA is 150~200 base pair fragments that are released by tumor cells into the blood representing a small fraction of the total cfDNA. Therefore, ctDNA carries tumor-specific mutations that are similar to the tumor itself [8,13]. It acts as a liquid biopsy once it consists of only a blood collection which can identify similar genetic alterations of the tumor itself. The tumor evolves, and many clinical decisions are made based on the tissue biopsy at diagnosis. The analysis of ctDNA opens the possibility to study the tumor in every time points of its progression, making it possible to obtain more information for clinical decisions.

ctDNA is a subset of total cfDNA and its detection varies [14] depending on tumor stage, vascularization, burden, apoptotic rate, and metastatic potential of the cancer cells, and the factors affecting the patient’s blood volume [15]. The ctDNA is released through lysis of necrotic and apoptotic cells; it may also be released from the digestion of tumor cells by macrophages or by direct secretion of DNA by tumor cells [16]. However, a recent study demonstrated that apoptosis is the main source of ctDNA since the induction of tumor apoptosis after chemotherapy treatment increases in ctDNA levels [17].

Detection of ctDNA has been challenging for three main reasons: Discrimination of ctDNA from normal cfDNA; the presence of extremely low levels of ctDNA; and the accurate quantification of the number of mutant fragments in a sample. Therefore, this discrimination is done by the recognition of mutations in the ctDNA. These somatic mutations, which are present only in the genomes of cancer cells, are not present in the DNA of normal cells [16,17]. Therefore, ctDNA can be useful to monitor tumor alterations, because it has a high turnover rate and is representative of the genomics of the tumor’s mutations, epigenetic changes, and microsatellite instability [18,19,20]. Although the use of ctDNA is a promising approach in cancer patients, it has some limitations: It is an expensive technique (mainly when using next-generation sequencing (NGS)); it does not provide information about morphological and tumor microenvironment status; in low tumor burden patients the chance of having informative results is low and it requires a previous knowledge of the targeted of interest mutation in most cases. Another limitation in the analysis of ctDNA is the extent to which the information obtained from the liquid biopsy sample reflects the tumor tissue; this is related to the fact that technical and biological factors can affect the concordance between tumor and plasma, generating false-negative and false-positive results in ctDNA analysis [21].

In breast cancer patients, plasma is the main fluid sample used for ctDNA analysis, while other body fluids are utilized for other cancer types [22] such as cerebrospinal fluid, urine, and saliva [23,24]. Next-generation sequencing (NGS) methods are used to examine ctDNA for the presence of gene variants, to detect early-stage disease, response to treatment, therapeutic resistance, and prognosis [25,26]. It also may detect disease progression and minimal residual disease after treatment [6]. It retains the genetic variant and epigenetic features of tumors, such as mutations, insertions, deletions, rearrangements, and DNA methylation of oncogenes or tumor suppressor genes, making it a potential biomarker [27]. Currently, NGS using molecular barcodes (unique molecular identifiers (UMIs)) can detect mutations at allele frequencies down to 0.1% and can be applied to highly sensitive detection of targeted ctDNA mutations. These methods include tagged-amplicon deep sequencing (TAm-seq), safe-sequencing system (Safe-SeqS), and cancer personalized profiling by deep sequencing (CAPP-Seq), with 97%, 98%, and nearly 100% sensitivity, respectively [28]. Methods using polymerase chain reaction (PCR) are also widely used, such as digital PCR (dPCR), which can detect minor allele frequencies less than 0.1% [28].

Whole genome sequencing (WGS) is usually used to get the whole genomic profile of tumor DNA including point mutations, rearrangements, and copy number variations (CNVs). It provides us with abundant information, but it is expensive and less sensitive. Whole exome sequencing (WES) may be an alternative. Nevertheless, both require high input sample volume, which hampers their application in routine testing and for screening and early diagnosis when the amount of ctDNA is low [28]. The development of these sequencing methods and novel exome sequencing kits led to the need for a robust statistical framework. According to the study published by Barbitoff, Y.A. et al. most of the observed bias in WES stems from mappability limitations of short reads, as well as exome probe design [29]. Due to the high complexity of the analysis, the lack of biological material, and the huge cost associated, neither WGS nor WES will be possible as routine molecular strategies used to analyze the ctDNA.

These advanced laboratory techniques have allowed for a daily collaboration between clinicians and laboratory teams since somatic mutations in tumors have gained a high clinical impact for patients due to the increasing targeted therapies available for some cancers. In 2016, the Food and Drug Administration (FDA) firstly approved the use of ctDNA as a liquid biopsy test for patients with stage IV non-small cell lung cancer (NSCLC) to check for epidermal growth factor receptor (EGFR) mutations [30]. This identification was often difficult in small lung tumor tissue samples available and repeating the lung biopsy was invasive for the patient. The identification of the mutation using ctDNA has allowed clinicians to overcome this difficulty and made it possible to treat patients with anti-EGFR targeted therapies available, today the standard of care. It has also allowed us to understand the resistance mechanisms of the tumors that stop responding to these treatments, and to select them for new therapies according to new mutations found in ctDNA. Spence, T. et al. analyzed the EGFR T790M status of 343 sequential patients with NSCLC using ctDNA collected during anti-EGFR treatment; 24% of the patients had the mutation and these patients were treated with osimertinib, a targeted drug approved in this scenario [31].

Therefore, a new paradigm of personalized medicine in which a targeted drug can be used in specific patients harboring a specific tumor mutation has emerged. Since this first clinical application approval, the use of ctDNA is being investigated in different cancer types, including breast cancer.

## 3. ctDNA—Clinical Application in Breast Cancer

Breast cancer is a complex disease whose molecular mechanisms are not completely understood. Developing targeted therapies is a promising approach in this area. Therefore, understanding the biological behavior of the tumor is the main challenge.

Liquid biopsies such as using ctDNA may help to overcome this, as they are a non-invasive method of monitoring the development of the disease during treatment and detecting emerging mutations [32]. They also allow tumor heterogeneity to be accessed, which can be related to acquiring therapy resistance [33]. Cancer is a heterogeneous disease, with different areas of the same tumor having different genetic profiles; likewise, heterogeneity exists between metastases within the same patient. A biopsy or tissue section from one part of a tumor may not represent the molecular intratumoral and intermetastatic heterogeneity [5,34,35]. As ctDNA from the primary tumor or metastasis is released into the blood, it may provide material for more comprehensive mutational profiling of the tumors in a less invasive way.

Although the first studies were in metastatic disease, the value of ctDNA in early disease has also been proven. Recent advances have also shown that ctDNA may also have a role in detecting residual disease after neoadjuvant chemotherapy and patient stratification due to genomic alterations, making it a valuable tool in locally advanced disease [6].

The concordance (same mutation status in plasma and tumor) between tumor tissue and ctDNA is being investigated, is not consistent across different studies, and has been established firstly in lung and colorectal cancers. In a study by Sung, J.S et al., 126 cases of non-small cell lung cancer patients were examined, and blood samples were analyzed for concordance of ctDNA and tumor tissue using ultra-deep sequencing and tissue genotyping. High concordance rates for *EGFR* mutations were observed [36]. In breast cancer, some studies have demonstrated concordance between breast cancer tissue and ctDNA. In a study conducted by Takeshita, T. et al., the authors compared *ESR1* mutation statuses of 35 cfDNA and matched tumor tissue in patients with metastatic breast cancer and found an overall concordance rate of 74.3% [37].

Woodhouse, R. et al. recently published their reports about clinical analytical validation of the FoundationOne^®^ Liquid CDx assay which is based on the first FDA-approved FoundationOne^®^ CDx tissue-based diagnostic tool analytically and clinically validated for solid tumors. The ability of the FoundationOne Liquid CDx assay to detect genomic alterations also detected in tumor tissue was demonstrated in this study. The test, recently approved by the FDA, consists of a cancer cfDNA-based comprehensive genomic profiling assay which targets 324 genes. It detects the major types of genomic alterations in addition to complex biomarkers such as microsatellite instability, tumor fraction, and blood tumor mutational burden [38].

The NILE study (Non-invasive versus Invasive Lung Evaluation; ClinicalTrials.gov; NCT03615443) is another FDA-approved study using cfDNA. The study has included 282 patients and has analyzed ctDNA in previously untreated metastatic NSCLC using this test. It allowed for the identification of tumor biomarkers with high concordance with tissue samples and in a more rapid way [39]. There are some assays using ctDNA approved for clinical use by FDA, and they have already been implemented in clinical practice. Table 1 describes the assays approved [40].

### 3.1. Early Diagnosis and Relapse

Early diagnosis of breast cancer is vital for reducing cancer-related mortality. It also allows for the selection of appropriate treatment approaches such as adjuvant therapy and therefore increases the chance of a patient’s survival. Clinically proven biomarkers that can be used to diagnose and guide patient management earlier in the course of the disease are not available. Serum-based protein biomarkers such as cancer antigen-125 (CA-125) and cancer antigen-15.3 (CA 15.3) are commonly used for monitoring breast cancer patients, but these proteins are also found in the serum of individuals without cancer; therefore, they are not useful for diagnosis [41]. Therefore, there are not sensitive biomarkers to detect distant metastases before they are radiological evident. Coombes, R.C et al. used the cfDNA of 49 patients collected after surgery and adjuvant therapy. The authors found that plasma ctDNA was detected before clinical or radiologic relapse in 16 of the 18 relapsed patients (sensitivity of 89%); metastatic progression was anticipated with a lead time of up to 2 years (median, 8.9 months; range, 0.5–24.0 months), providing a good opportunity for clinical intervention [42].

Very low levels of ctDNA are usually not detectable in early disease [42]. Therefore, in the setting of early breast cancer, the clinical utility of ctDNA-based liquid biopsy remains unclear, as the percutaneous biopsy of breast tumors continues to be preferred for diagnosis. Nevertheless, a blood test (CancerSEEK) has already been studied for early detection of some cancers such as ovary, liver, stomach, pancreas, esophagus, colon, rectum, lung, and breast cancer [43]. According to this study, this multi-analyte blood test was able to detect some cancers through the determination of mutations and circulating proteins using the cfDNA. The median sensitivity of the test was 70% among the cancer types studied, but it was lower for breast cancer tumors (about 33%) [43].

Some studies have shown that the fraction of ctDNA detected in a breast cancer patient in early disease is directly correlated with disease stage and is significantly lower at earlier stages of the disease [44]. As an example, Board, R.E et al. showed that the mutation rates observed in ctDNA within genes such as PIK3CA were much higher in advanced-stage breast tumors compared to stage I and II tumors [45]. The authors also found a concordance of 95% between ctDNA and tissue samples of the tumors.

Several studies have already examined the potential of ctDNA for early detection of breast cancer at early stages [46,47]. In a study conducted by Phallen, J. et al., an evaluation of 200 patients with colorectal, breast, lung, or ovarian cancer detected somatic mutations in the plasma of 71%, 59%, 59%, and 68%, respectively, of patients with stage I/II disease. Analyses of mutations in blood using ctDNA revealed high concordance with alterations in the tumors of these patients (72%) [46].

A prospective and multicenter study of 101 women with early-stage breast cancer using circulating tumor DNA mutation tracking found that detection of circulating tumor DNA during follow-up had a median lead-time of 10.7 months compared with clinical relapse, anticipating relapse in all breast cancer subtypes [48]. This anticipation of clinical relapse may lead us to take clinical measures that allow for the detection of early recurrence and properly managing it.

Kim, C. et al. found that the low-level expression of estrogen receptor 1 (ESR1) in breast cancer tissue samples is correlated with endocrine therapy resistance in estrogen receptor (ER)-positive primary breast cancer and is associated with the treatment outcome [49]. *ESR1* mutations can also be identified using ctDNA analysis and predict resistance to endocrine therapy in early disease. Nevertheless, they are rarely detected during adjuvant treatments [50].

Recently, a prospective study conducted by Liu M.C, et al. investigated the ability of target methylation analyses of cfDNA of different cancer types to detected and localize different cancer types. The authors concluded that cfDNA sequencing and analysis of methylation patterns detected more than 50 cancer types across different stages, including breast cancer [51].

Therefore, although not validated for diagnosis, ctDNA may play an important role in early breast cancer evaluation and decision making.

### 3.2. Metastatic Disease

Measuring treatment response in patients with metastatic breast cancer is usually done by clinical evaluation, assessment of CA15.3 levels, and radiographic imaging. However, serial radiographic imaging is often inconclusive, and CA 15.3 fluctuations do not necessarily reflect tumor response or progression [52].

No marker for monitoring therapy responses in patients with metastatic breast cancer has yet reached wide clinical use, making it difficult for clinicians to anticipate a disease progression. The use of ctDNA in breast cancer was first established in metastatic disease, as tumor burden was related to the total amount of ctDNA [53].

In metastatic breast cancer, ctDNA has shown promising results to monitor disease as reported by Dawson, S.J et al. [54]. In this study, the authors compared the radiographic imaging of tumors with ctDNA, CA 15-3, and circulating tumor cells in 30 women with metastatic breast cancer who were receiving systemic therapy. They concluded that ctDNA levels showed a greater dynamic range and correlation with changes in tumor burden than CA 15-3 or circulating tumor cells. The authors mainly used the detection of mutations in the genes PIK3CA and TP53 as surrogates for ctDNA. At the time, there was no mutation target therapy approved for metastatic breast cancer.

Some clinical studies have investigated ctDNA mutations associated with targeted therapy response in patients with HER2-positive breast cancer and endocrine therapy response in patients with ER-positive metastatic breast cancer [55,56]. These studies showed that ESR1 mutations normally arise following the treatment of metastatic disease and can predict resistance to endocrine therapy with aromatase inhibitors [55]. Acquired ESR1 missense alterations occur in about 30% of patients who have received prior endocrine therapies and are associated with an aggressive clinical phenotype of the disease [56]. Fribbes, C. et al. conducted a prospective–retrospective analysis, assessing ESR1 mutations in available archived plasma from the Study of Faslodex vs. Exemestane with or without Anastrozole (SoFEA) and Palbociclib Combined with Fulvestrant in Hormone Receptor-Positive HER2-Negative Metastatic Breast Cancer after Endocrine Failure (PALOMA3) trials. The authors concluded that ESR1 mutation analysis in plasma after progression can be a useful tool to guide the clinician’s choice for subsequent endocrine therapies [56].

Detection of ctDNA nucleotide alterations to assess response to anti-HER2-targeted therapies has also been investigated [57]. As reported in Ye, Q. et al. study, 46 genes were detected from an assessment of 486 single-nucleotide variants; only 7 genes considered relevant to targeted therapy resistance were detected in the treatment-resistant group. In addition, two patients in whom HER2 p.S855I mutations were detected had benefited from anti-HER2 therapy. Therefore, the authors concluded that targeted NGS of ctDNA has a potential clinical utility to detect biomarkers from HER2-targeted therapies [57]. In a recent study from Guan, X. et al., the authors found that the level of HER2 amplification in ctDNA by NGS had a high concordance with the primary tumor (approximately 80%); moreover, HER2 copy numbers showed fluctuations during HER2-targeted therapies, and patients with positive levels after 6 weeks of treatment showed a reduction in progression-free survival [58]. With the widely recommended use of anti-HER2 target drugs for treatment of HER2-positive breast cancer patients in metastatic disease due to the benefits in overall survival and progression-free survival, it is important to explore the molecular mechanisms behind the good results of this therapy and monitor them. On the other hand, it is important to understand the reason why some tumors do not respond to anti-HER2 targeted therapies to be able to investigate the mechanisms of tumor resistance in this scenario and overcome them.

Recently, targeted therapy for PIK3CA mutations has emerged. These mutations are found in 20–30% of breast cancer patients [59,60]. Alpelisib is a targeted drug approved for treating metastatic breast cancer patients with positive endocrine receptors and PIK3CA mutations. It is an α-specific PI3K inhibitor that selectively inhibits p110α approximately 50 times as strong as other isoforms [59]. In a phase 3 study (SOLAR 1 trial), alpelisib plus fulvestrant were compared with placebo plus fulvestrant in patients with HR-positive and HER2-negative advanced breast cancer who had previously been treated with endocrine therapy. The results showed that treatment with alpelisib and fulvestrant prolonged progression-free survival among these patients’ population [60]. According to this trial, the use of alpelisib was approved in patients with metastatic HR-positive, HER2-negative, and PIK3CA-mutated tumors. The use of ctDNA to identify PIK3CA mutations was also investigated in a subgroup analysis of SOLAR 1 trial by Juric, D. et al. and the treatment showed consistent clinically meaningful treatment benefit for patients with ctDNA mutant status [61]. It made it possible to use a more comfortable and less invasive tool to detect a tumor mutation for which a targeted therapy is available.

Breast cancer disease carrying a BReast CAncer gene (BRCA) mutation also represents an intriguing scenario. Poly (ADP-ribose) polymerase (PARP) inhibitors such as olaparib and talozaparib are approved in metastatic breast cancer patients carrying a germline BRCA1 or -2 mutation and HER2-negative tumors. These drugs are used taking into account the rationale of synthetic lethality mechanism, which means two conditions that independently would not cause cell death when used in combination are lethal. In patients carrying a germline BRCA1 or BRCA2 mutation, homologous recombination pathway is defective; the use of PARP inhibitors to hamper the base excision repair (BER) mechanism causes cell damage and consequently death [62]. In OlympiAD and EMBRACA trials, olaparib and talozaparib, respectively, showed a median progression-free survival and response rate significantly longer than in the standard chemotherapy group in this subgroup of germline BRCA1 or BRCA2-mutated patients [62,63,64]. Therefore, ctDNA-based assessment of somatic BRCA mutations using ctDNA could potentially expand the cohort of patients treatable with PARP inhibitors in the future [65].

More recently the resistance mechanisms of cyclin inhibitor drugs using ctDNA have been studied. A study from Condorelli R. et al. has shown a retinoblastoma gene (Rb) mutation emerging after cyclin inhibitors treatment and failure. The mutation was detected in the ctDNA of an ER-positive, HER2-negative metastatic breast cancer patient. Cyclin inhibitors are approved in metastatic ER-positive and HER2-negative breast cancer patients in first and second therapy lines. They act by inhibiting the cyclin D1-CDK4/6-retinoblastoma pathway [66,67].

There are some clinical trials currently addressing the question of using ctDNA to select targeted therapies for breast cancer patients. The PLASMAmatch trial is a phase 2 trial that included 1051 patients, with ctDNA available for 1034 of them. Patients were divided in different treatment cohort groups according to the mutation identified and target therapy used. The authors found that ctDNA testing was highly accurate, and showed high sensitivity for mutations identified in advanced breast cancer tissue biopsies. ctDNA was able to identify patients with important targetable mutations and therefore it could be used in routine clinical practice [68].

As described above, liquid biopsies are being investigated and applied in different cases mainly in metastatic breast cancer, with targeted therapies for specific mutations being constantly approved for this subgroup of patients. This application in clinical practice is increasing and has led to a constant need of molecular laboratories to develop new strategies to provide quick and quality molecular results to medical oncologists not only using ctDNA, but also tumor tissue. These results are currently fundamental to decision making in clinical practice; therefore, improving investigation and sharing results in this area is mandatory.

### 3.3. Locally Advanced Breast Cancer: Detecting Residual Minimal Disease

Patients with locally advanced breast cancer are commonly treated with neoadjuvant therapy (NAT) to reduce cancer size and study the treatment response to chemotherapy. 

In breast cancer, ~30% of patients treated with NAT achieve pathological complete responses (pCR) with no histological evidence of invasive tumors in the resected breast tissue and lymph nodes [67]. Achieving pCR is correlated with a good prognosis of the disease. Patients with HER2-positive and triple-negative breast cancer tumors are the best candidates for neoadjuvant chemotherapy as they have the highest probability of achieving pCR [69]. Analysis of gene expression changes in ctDNA of sequential patient samples collected before and during treatment may be a promising way to analyze the molecular changes that occur during treatment in real-time. Comparing samples from patients who have a complete response to neoadjuvant treatment to samples from those who do not have it can provide a better understanding of the resistance mechanisms of the tumor, allowing for the definition of a better therapy strategy for the patients. Nevertheless, detection of ctDNA after NAT has been challenging in patients, even when the residual disease is observed in the surgery. Recent studies have found that ctDNA becomes undetectable in more than 90% of patients during NAT [70]. Nevertheless, no correlation has been established between pCR and ctDNA.

A recent study showed that after completion of neoadjuvant therapy, ctDNA concentrations were lower in patients who achieved pCR compared to patients with residual disease. Further, patients with pCR showed a larger decrease in ctDNA concentrations during neoadjuvant therapy [71]. Previously, some studies already suggested that during NAT, ctDNA levels decreased and minimal residual disease of the tumor was undetectable after the surgery. A slow decrease of ctDNA levels during NAT was associated with poorer survival [72].

A recent study from Radovich, M. et al. showed that detection of ctDNA in patients with early-stage triple-negative breast cancer after neoadjuvant chemotherapy was independently associated with disease recurrence. The authors used the samples of a cohort of 196 patients with locally advanced breast cancer submitted to neoadjuvant chemotherapy. At 24 months, disease-free survival probability was 56% for ctDNA-positive patients compared with 81% for ctDNA-negative patients [73].

Liquid biopsy is a promising option for detecting driver somatic mutations, and the low ctDNA levels found in these early stages represent a challenge for sequencing techniques. More studies are needed in this specific clinical scenario to overcome this difficulty and to achieve a prognostic molecular tool for these patients. This may allow us, in the future, to optimize the clinical follow-up of these patients and predict who are more likely to have recurrence of the disease.

## 4. Conclusions

ctDNA sequencing analysis is an important molecular tool option to inform on tumor mutational and molecular landscapes in a less invasive way. It could be used in early-stage disease, allowing for the detection of early recurrences; however, it is also a valuable method to longitudinally monitor tumors’ genomic profiles and detect the emergence of genetic alterations in the metastatic scenario, anticipating clinical symptoms or radiological evidence of disease progression. Additionally, it allows for a better selection of targeted therapies and provides a more comprehensive photogram of tumor heterogeneity. Consequently, ctDNA analysis may guide clinical decision-making in the precision medicine era (Table 2).

## Figures and Tables

**Table 1:** Food and Drug Administration (FDA)-approved liquid biopsies assays.

Liquid Biopsy Assay	Clinical Application	Genes Analyzed
FoundationOne ^®^ Liquid CDx assay	NSCLC, mCRPC	70 genes + MSI-H (BRCA 1, 2, EGFR)
Guardant360 ^®^ CDx assay	NSCLC, pan-cancer	70 genes using NGS
Therascreen ^®^ (Qiagen) PI3KCA	Breast cancer	11 mutation in PIK3CA gene
EpiproColon ^®^	Colorectal cancer	PCR, methylation
Cobas ^®^ EGFR mutation test (Roche)	NSCLC	EGFR variants
In Vision First-Lung ^®^	NSCLC	37 genes NSCLC
Oncobeam Lung-1 ^®^	NSCLC	EGFR
Oncobeam Lung-2 ^®^	NSCLC	EGFR, KRAS, BRAF
Oncomine ^®^ (Thermo Fisher Scientific)	Breast, lung, colon cancer, pan-cancer	52 genes cancer assay
TS0500 ctDNA ^®^ (Illumina)	Pan-cancer	500+ genes
Avenio ctDNA ^®^ (Roche)	Breast, lung, colorectal, gastric, melanoma, pancreatic, ovarian, glioma, thyroid cancers	17 genes

Non-small cell lung cancer (NSCLC); metastatic castration resistant prostate cancer (mCRPC); microsatellite instability (MSI-H).

**Table 2:** Clinical utility of using ctDNA in different breast cancer scenarios.

Breast Cancer Scenario	Clinical Use	Assay Used	Findings	Reference
Early-stage disease	Early detection	CancerSEEK	Detect cancers through the determination of mutations using the cfDNA. The median sensitivity of the test was 70% among the cancer types studied; 33% in breast cancer.	[43]
	Anticipating relapse	Ultra deep sequencing	ctDNA was detected before clinical or radiologic relapse in cancer patients (sensitivity of 89%).	[42]
		dPCR analysis of ctDNA	Detection of ctDNA during follow-up is associated with high risk of relapse.	[48]
	Treatment resistance	dPCR analysis of ctDNA	*ESR1* mutations can predict resistance to endocrine therapy in early disease.	[50]
Metastatic disease	Monitoring disease	dPCR analysis of ctDNA	The ctDNA levels showed a greater dynamic range, and correlation with changes in tumor burden, than CA 15-3 or circulating tumor cells in patients with breast cancer receiving therapy.	[54]
	Treatment resistance	NGS and dPCR analysis of ctDNA	ESR1 mutations can predict resistance to endocrine therapy; ESR1 mutation analysis in plasma after progression can be a useful tool to guide the clinician’s choice for subsequent endocrine therapies.	[55,56]
	Selecting targeted therapies	dPCR analysis of ctDNA	According to the SOLAR 1 trial, alpelisib was approved in patients with PI3KCA mutation; the use of ctDNA to identify PI3KCA mutation was validated.	[60,61]
		PLASMAmatch	ctDNA was able to identify patients with important targetable mutation.	[68]
Locally advanced disease	Detecting minimal residual disease	Dropped dPCR; Targeted digital sequencing (TARDIS)	ctDNA concentrations were lower in patients who achieved pCR compared to patients with residual disease; slow decrease of ctDNA levels during NAT was associated with poorer survival.	[71,72]
		Foundation One^®^ Liquid Assay	Detection of ctDNA in patients with early-stage triple-negative breast cancer after neoadjuvant chemotherapy was independently associated with disease recurrence.	[73]
